# Recreational cannabis legalization and immigration enforcement: a state-level analysis of arrests and deportations in the United States, 2009–2020

**DOI:** 10.1186/s12889-024-18334-y

**Published:** 2024-04-01

**Authors:** Emilie Bruzelius, Silvia S. Martins

**Affiliations:** Substance Use Epidemiology Training Program (SAETP), Department of Epidemiology, Joseph L. Mailman School of Public Health, Columbia University, 722 W. 168th St. Suite 511, 10032 New York, NY USA

**Keywords:** Policy, Immigration, Migration, Cannabis, Law enforcement

## Abstract

**Background:**

Recreational cannabis laws (RCL) in the United States (US) can have important implications for people who are non-citizens, including those with and without formal documentation, and those who are refugees or seeking asylum. For these groups, committing a cannabis-related infraction, even a misdemeanor, can constitute grounds for status ineligibility, including arrest and deportation under federal immigration policy—regardless of state law. Despite interconnections between immigration and drug policy, the potential impacts of increasing state cannabis legalization on immigration enforcement are unexplored.

**Methods:**

In this repeated cross-sectional analysis, we tested the association between state-level RCL adoption and monthly, state-level prevalence of immigration arrests and deportations related to cannabis possession. Data were from the Transactional Records Access Clearinghouse. Immigration arrest information was available from Oct-2014 to May-2018 and immigration deportation information were available from Jan-2009 to Jun-2020 for. To test associations with RCLs, we fit *Poisson* fixed effects models that controlled for pre-existing differences between states, secular trends, and potential sociodemographic, sociopolitical, and setting-related confounders. Sensitivity analyses explored potential violations to assumptions and sensitivity to modeling specifications.

**Results:**

Over the observation period, there were 7,739 immigration arrests and 48,015 deportations referencing cannabis possession. By 2020, 12 stated adopted recreational legalization and on average immigration enforcement was lower among RCL compared to non-RCL states. In primary adjusted models, we found no meaningful changes in arrest prevalence, either immediately following RCL adoption (Prevalence Ratio [PR]: 0.84; [95% Confidence Interval [CI]: 0.57, 1.11]), or 1-year after the law was effective (PR: 0.88 [CI: 0.56, 1.20]). For the deportation outcome, however, RCL adoption was associated with a moderate relative decrease in deportation prevalence in RCL versus non-RCL states (PR: 0.68 [CI: 0.56, 0.80]; PR 1-year lag: 0.68 [CI: 0.54, 0.82]). Additional analyses were mostly consistent by suggested some sensitivities to modeling specification.

**Conclusions:**

Our findings suggest that decreasing penalties for cannabis possession through state RCLs may reduce some aspects of immigration enforcement related to cannabis possession. Greater attention to the immigration-related consequences of current drug control policies is warranted, particularly as more states weigh the public health benefits and drawbacks of legalizing cannabis.

**Supplementary Information:**

The online version contains supplementary material available at 10.1186/s12889-024-18334-y.

## Background

A growing number of people in the United States (U.S.) live in a jurisdiction where cannabis use is legal. As of January 2023, 38 states and the District of Columbia have legalized cannabis use for medical purposes. A further 27 states have also legalized or decriminalized adult possession of cannabis for recreational purposes. Despite increasing legalization, however, arrests for low-level cannabis offenses (e.g., age restrictions, public consumption) remain prevalent, even in places with legalization. For example, there were more than 20,000 misdemeanor cannabis arrests in California in the six years after recreational cannabis was legalized in 2016 [1]. Nationally, of the more than 500,000 cannabis arrests in 2019, over 91% were for possession [2]. 

Persistently high rates of cannabis arrests in the U.S. have important social justice and health equity implications, especially for populations disproportionately affected by cannabis prohibition. For example, compared to White people, Black and Latinx people report similar or lower rates of cannabis use, but dramatically higher rates of cannabis-related criminal-legal system contact, including arrests, prosecutions, convictions and incarcerations [3–5]. A prominent argument in favor of state cannabis law reform has been that legalization should reduce some of the racialized harms associated with cannabis enforcement [6]. However, studies to date suggest a more nuanced picture. In the U.S. and Canada, recreational cannabis laws (RCLs) have been associated with reductions in total cannabis arrests and racialized arrest disparities measured on the absolute scale [7–13]. When measured on the relative scale, however, studies have found that racialized disparities commonly persist or even increase following law adoption, as the harms associated with cannabis enforcement continue to be disproportionately concentrated among racialized groups [8, 9, 11, 14]. In addition, because limited research has evaluated the potential downstream consequences of arrests and arrest disparities, including on convictions, criminal record expungement, and other related outcomes, the wider effects of legal changes are still unclear [15–17]. 

Another important but less commonly discussed equity consideration for cannabis legalization is its potential impacts on immigration justice and immigrant health disparities [6]. Because cannabis remains illegal at the federal level, cannabis infractions, even for minor or civil offences, and otherwise ‘legal’ cannabis-related conduct, can have severe repercussions for people who are not US citizens, including temporary or permanent residents, dreamers and those granted asylum [18]. Under federal policy, a conviction, charge, or admission of simple cannabis possession is considered by U.S. Immigration and Customs Enforcement (ICE) as sufficient grounds for status ineligibility, arrest, detention, or deportation, as is employment in the cannabis industry [19]. Further, as immigration authorities often work with police to identify people with drug-related violations for deportation, cannabis prohibition has been theorized as a primary mechanism of the arrest-to-deportation pipeline [20]—a term describing the series of mutually reinforcing policies and practices that funnel predominantly lower income people of color who are non-citizens from contact with law enforcement into deportation proceedings [21]. 

Despite this interplay between criminal-legal and immigration system policies, existing research has yet to consider cannabis legalization as a potential structural lever for reducing immigration enforcement and its adverse health consequences. Research has shown that immigration-related stressors (e.g., fear of deportation), exposure to exclusionary immigration policies (e.g., requirements for carrying registration documents) and direct experiences of arrest or deportation, are associated with numerous adverse mental and physical health conditions [22]. Moreover, emerging research indicates that these health detriments can extend beyond non-citizens themselves, to their family members and broader communities [23]. 

Two countervailing pathways are relevant to anticipating the potential immigration implications of RCL adoption. First, RCLs could lead to potential decreases in the overall number of cannabis-related arrests or convictions, and therefore cannabis-related immigration enforcement. A second possibility, however, is that state adoption of RCLs might lead more people who are non-citizens to reasonably but falsely assume that federal immigration status is unaffected by cannabis use permissible under state law—potentially leading to increases in immigration enforcement. This second possibility is noteworthy in that recent evidence suggests that while immigrant populations remain less likely than non-immigrants to engage in any substance use, [24, 25] cannabis use has increased significantly among immigrant and foreign-born adults in the past decade [26]. In the present study, we therefore tested whether the adoption of a state-level RCL was associated with changes in the monthly, state-level prevalence of immigration arrests and deportations related to cannabis possession between 2009 and 2020.

## Methods

### Study population

Data on immigration arrests and deportations were obtained from the Transactional Record Access Clearinghouse (TRAC) at Syracuse University. TRAC obtains data through freedom of information act fillings and litigation and makes this information viewable to the public through a series of web tools, and downloadable by subscription. TRAC records are increasingly used in public health research to measure various facets of immigration enforcement [27–29]. 

### Outcomes

Although TRAC provides information on multiple measures of immigration enforcement activity (i.e., detainers, detentions, etc.), not all data are complete. We therefore concentrated on the two outcomes—arrests (Oct-2014 to May-2018) and deportations (i.e., ‘removals;’ Jan-2009 to Jun-2020)—for which information was consistently available at the month-year level, and where references to a cannabis-related offence were included in the data. Arrests refer specifically to those made by ICE agents in response to an immigration offence—though arrests often occur in cooperation with federal, state, or local law enforcement agencies or via delegation to non-ICE law enforcement through the 287(g) program. Counts excluded arrests made at the US-Canada and US-Mexico borders by Customs and Border Protection. Because our focus was on RCLs, we concentrated on arrests and deportations noting cannabis possession as part of underlying immigration offense based on the National Crime Information Center (NCIC) coding system. We included records if they specified that the individual was convicted of, charged with, or held in custody for reasons related to ‘marijuana possession.’ Counts excluded offences referencing sales, smuggling, or broadly referencing ‘marijuana’ or ‘drugs,’ but not specifying cannabis possession directly. To generate prevalence rates from counts, we used the total annual state population estimates from the National Center for Health Statistics as denominators or as offsets in models [30]. 

### Exposures

Cannabis law data, including dates for the primary exposure—RCLs—was based on prior literature, as well as information from the Alcohol Policy Information System, Cannabis Policy Database and ProCon.org and are presented in Table 1 [11, 31–35]. From these sources, we also captured covariate legal information, including state medical cannabis legalization and decriminalization (removal of criminal penalties), inclusive of depenalization (removal of all penalties). Because we were primarily interested in the legal status of cannabis within a state, as opposed to overt access to a legal cannabis supply (via dispensaries or a home cultivation provision), we coded law exposures based on the date that a RCL became effective, rather than passage or commercial sales implementation dates.

**Table 1:** State cannabis law effective dates

State	RCL	MCL	Decriminalization
Alaska	2/24/15	Pre-2009	Pre-2009
Arizona		2010	Pre-2009
Arkansas		2016	
California	11/9/16	Pre-2009	Pre-2009
Colorado	12/10/12	Pre-2009	Pre-2009
Connecticut		2012	2011
Delaware		2011	2015
District of Columbia	2/26/15	2010	
Florida		2017	
Hawaii		Pre-2009	2020
Illinois	1/1/20	2014	2016
Kansas			2017
Louisiana		2016	2021
Maine	1/30/17	Pre-2009	Pre-2009
Maryland		2014	2014
Massachusetts	12/15/16	2013	2008
Michigan	1/1/20	Pre-2009	
Minnesota		2014	Pre-2009
Mississippi			Pre-2009
Missouri		2018	2017
Montana		Pre-2009	2017
Nebraska			Pre-2009
North Carolina			Pre-2009
Nevada	1/1/17	Pre-2009	
New Hampshire		2013	2017
New Jersey		2010	
New Mexico		Pre-2009	2019
New York		2014	Pre-2009
North Dakota		2016	2017
Ohio		2016	Pre-2009
Oklahoma		2018	
Oregon	3/29/16	Pre-2009	Pre-2009
Pennsylvania		2016	
Rhode Island		Pre-2009	2013
Utah		2018	
Vermont	7/1/18	Pre-2009	2013
Washington	12/6/12	Pre-2009	
West Virginia		2018	

RCL: recreational cannabis law, MCL: medical cannabis law. Dates refer to the date that the law became effective rather than the data it was passed or enacted

### Covariates

In addition to other state cannabis policies (medical legalization, decriminalization), control variables included time-varying state-level factors that we expected to be associated with RCL adoption and immigration enforcement. Specifically, we controlled for census-derived demographic factors including median household income, share of the population identifying as BIPOC (Black, Indigenous, other people of color including Hispanic, Asian), and share of the population not proficient in English. Sociopolitical factors included the prevalence of police personnel (including nonsworn personnel) [36] and governor’s political party affiliation [37]. We also controlled for seasonality (Jan-Mar, Apr-Jun, Jul-Sep, Oct-Dec) given that enforcement patterns might vary by season by location. Lastly, we included an indicator to capture implementation of the ICE Secure Communities program which was rolled out across US states between 2008 and 2013. This program requires that certain types of contacts with law enforcement trigger automatic immigration database checks and its implementation has been associated with significant increases in immigration arrests and deportations [38, 39]. 

### Statistical analysis

To test associations between RCLs and changes in arrests and deportations related to cannabis possession we began by described overall outcome trends by plotting the unadjusted prevalence of monthly cannabis possession arrests and deportations over time, stratified by legalization status at the end of the observation period. In addition, we examined the distribution of state-level demographic and sociopolitical variables, also by legalization status. We then used a series of separate regression models to test associations between RCLs and both outcome measures. In addition to state and time-based (month-year) fixed effects, our primary specification included demographic and sociopolitical variables, however we additionally present unadjusted model results for comparison. Given the count nature of the data, we specified models used *Poisson* regression and including population offsets [40]. Additional sensitivity models tested other specification as described in further detail below. In terms of the RCL exposure, we assumed that any effect of law adoption on immigration enforcement patterns would be relatively immediate. However, because there may be some degree of lag time between when a RCL goes into effect and when it might reasonably impact on the ground immigration enforcement actions, we additionally conducted analyses including a 1-year RCL lag to probe for potential differences by law timing.

### Sensitivity analyses

We conducted 4 sets of supplemental analyses to test assumptions and to evaluate the overall robustness of the results. First, we conducted an event study to help evaluate the assumption that states without RCLs serve a poor comparison for what would have happened in RCL states in the absence of legalization. To test this assumption, we re-ran the primary adjusted regression models replacing the binary RCL variable with a series of RCL leads and lags to test for differences in outcome trends between RCL and non-RCL states, prior to RCL adoption. Second, we replaced the estimates of the total state population with estimates of the unauthorized immigrant population within a state using data from the Pew Research Center [41]. Third, for the arrest outcome, we excluded two states, Colorado and Washington, that had already adopted an RCL prior to the start of the observation period in October 2014 (Table 1) given that the inclusion of such ‘always treated’ units can potentially bias results [42]. Fourth, given that the COVID-19 pandemic likely altered law enforcement and immigration patterns, potentially in ways unrelated to cannabis laws adoption, we re-ran the primary analyses excluding data from 2020. Finally, we also re-ran the primary models using a quasi-*Poisson* specification to evaluate potential overdispersion (i.e., outcome variance greater than the mean) in the models.

Because all data were publicly viewable, de-identified, and at the group-level (state), analyses were considered non-human subjects research and exempt from institutional board review. Analyses were conducted using R version 4.3.2. (R Foundation for Statistical Computing).

## Results

Of the nearly half a million immigration arrests that occurred between Oct-2014 and May-2018, 7,739 specifically referenced cannabis possession. Similarly, of the more than 5 million deportations between Jan-2009 and Jun-2020, 48,015 referenced a cannabis possession. By the end of the full observation period in Jun-2020, 12 states had adopted RCLs, including 2 states (WA and CO) that adopted legalization prior to the 2014—the start of the study period for the arrest outcome. All states with recreational legalization had previously adopted medical legalization and some had further previously adopted decriminalization (Table 1).

**Fig. 1:**
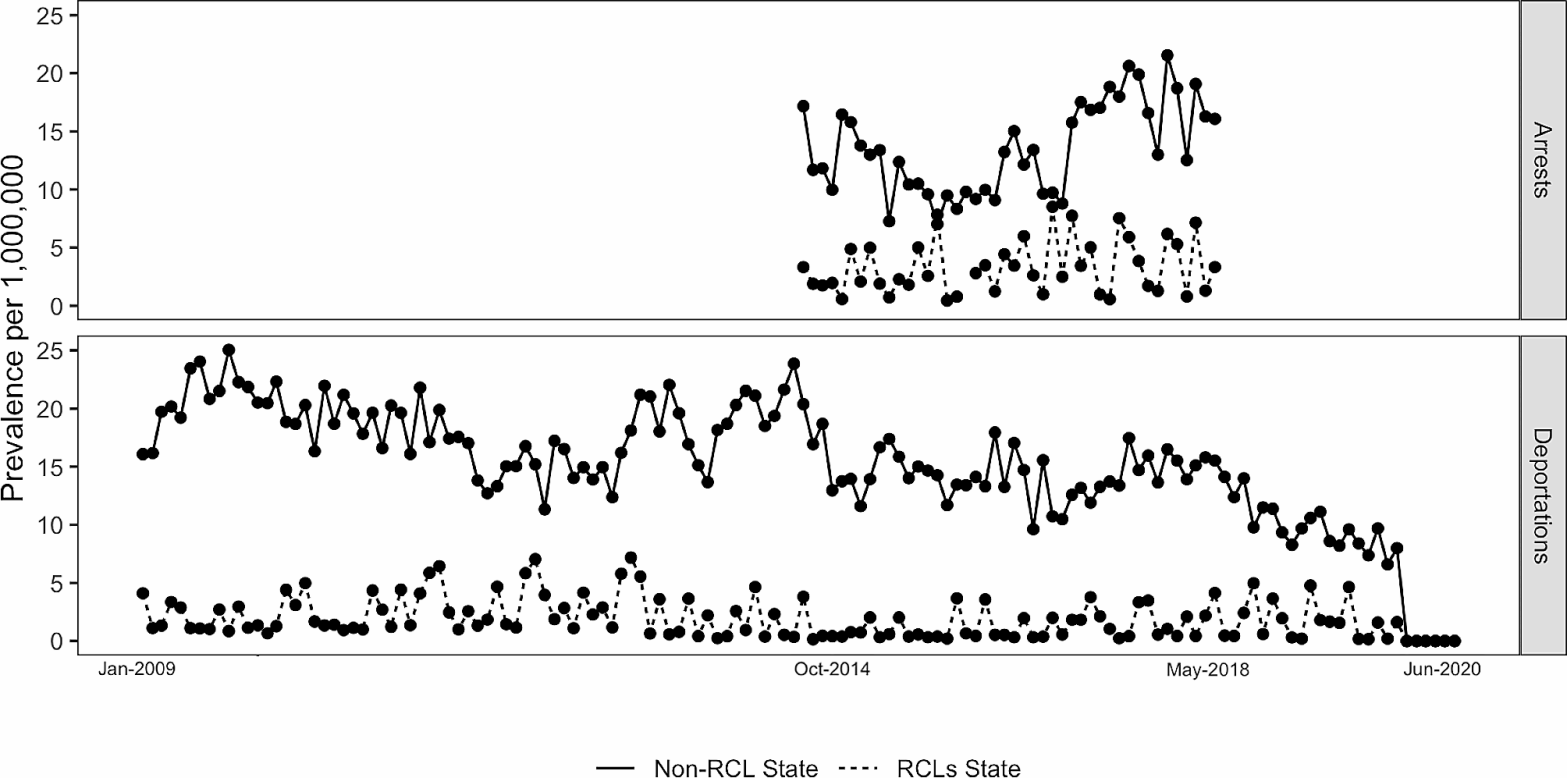
Trends in cannabis possession-related arrests and deportations by recreational cannabis legalization (RCL) status, 2009–2020. The upper panel displays the average prevalence of immigration arrests specifying cannabis possession (per 1,000,000 state population) disaggregated by whether the state adopted an RCL by the end of the observation period in 2019; the bottom panel displays trends in deportation. Dashed lines represent RCL states whereas solid lines represent states without RCL. Arrest data were only available for the period from Oct 2014 to May 2018

Figure 1 displays the trends in immigration arrests and deportations referencing cannabis possession between Jan-2009 and Jun-2020. Overall, prevalence rates of both measures were lower among RCL compared to non-RCL states. There was a notable drop in deportations in 2020, coinciding with the onset of the COVID-19 pandemic. Arrest trends in both legalization and non-legalization states were relatively similar, and generally stable over the period. For the deportation outcome, trends suggested that the overall prevalence of deportations decreased between 2009 and 2020 (Fig. 1). In terms of demographic and sociopolitical factors, states that adopted RCLs tended to have a higher median household income (approximately $72,405 verses $59,417), a lower prevalence of police personnel (6.41 versus 9.55 per 1,000 population), and more commonly had a governor affiliated with the democratic party than non-legalization states (Table 2).

**Table 2:** State demographic and sociopolitical factors by recreational cannabis legalization status, 2009–2020

	Non-RCL states (*n* = 6,454year-monthsof observation)		RCL states (*n* = 550year-monthsof observation)	
Monthly cannabis possession immigration arrests per 1,000,000 population, [mean (SD)]	0.32	(0.66)	0.39	(1.06)
Monthly cannabis possession immigration deportations per 1,000,000 population, [mean (SD)]	0.35	(1.35)	0.13	(0.54)
Share of population identifying as BIPOC, [mean (SD)]	0.30	(0.16)	0.36	(0.17)
Share of population not proficient in English, [mean (SD)]	0.04	(0.03)	0.04	(0.02)
Median household income, [mean (SD)]	59,417.23	(9,501.41)	72,405.03	(8,758.01)
Police personnel per 1,000 population,^A^ [mean (SD)]	9.55	(5.62)	6.41	(4.82)
Governor’s political party [n (%)]				
Democrat	2704	(41.89)	396	(72.00)
Republican or Independent	3750	(58.10)	154	(28.00)

RCL: Recreational cannabis law

^A^Includes nonsworn personnel

Table 3 shows the primary results from unadjusted and adjusted models testing associations between RCL adoption and immigration enforcement prevalence. In the primary adjusted models, RCLs were associated with an approximately 16% immediate relative decrease in arrests but with confidence intervals that crossed the null, (Prevalence Ratio [PR]: 0.84; 95% Confidence Interval [CI]: 0.57, 1.11). Models incorporating a 1-year lag were also negative, but showed slightly attenuated associations, again with a confidence intervals that crossed the null (PR [1-year lag]: 0.88; CI: 0.56, 1.20). For deportations, in the primary models we observed a moderate negative association with RCLs, indicating an immediate relative decrease in deportations following law adoption (PR: 0.68 CI: 0.56, 0.80). Results were also consistently negative after incorporating the 1-year RCL exposure lag (PR: 0.68; CI: 0.54, 0.82).

**Table 3:** Association between recreational cannabis legalization and immigration arrests and deportations related to cannabis possession, 2009–2020

	Cannabis Arrests (October 2014-May 2018, *n* = 2,244)						Cannabis Deportations (January 2009-June 2020, *n* = 7,004)					
	Unadjusted			Adjusted			Unadjusted			Adjusted		
	PR	95% CI		PR	95% CI		PR	95% CI		PR	95% CI	
RCL (immediate)	0.76	0.52	0.99	0.84	0.57	1.11	0.66	(0.55,	0.76)	0.68	(0.56	0.80)
RCL (1-year lag)	0.83	0.53	1.13	0.88	0.56	1.20	0.71	(0.59,	0.84)	0.68	(0.54,	0.82)

Recreational cannabis legalization status was operationalized as a binary variable capturing whether a state had a recreational cannabis law in place in the month-year. Models were implemented using a *Poisson* specification and adjusted models included controls for state, month-year, presence of a medical cannabis and decriminalization law, governor’s political party, prevalence of police officers, state median household income, percent of the state population identifying as BIPOC, percentage of the state population not proficient in English and including logged state population as an offset

Sensitivity and model robustness checks were as follows. First, Supplemental Fig. 1 and Supplemental Fig. 2 display results from the event study. They suggest that the estimated associations were not predominantly driven by differences in pre-RCL trends between RCL and non-RCL states. However, both sets of results also showed wide confidence intervals in the post-RCL period. While few of the RCL associations with arrests were estimated with precision in the primary analysis (which was confirmed in the event-study), there was also significant imprecision for the deportation outcome suggesting that findings should be interpreted with caution. Second, when we replaced the state population estimates with estimates of the unauthorized immigrant population in a state, associations were slightly attenuated but did not qualitatively change (Supplemental Table 1). Third, when we excluded the always treated states (CO and WA) from the arrest models there were no changes with respect to immediate associations with RCL adoption (PR: 0.84; CI: 0.56, 1.11, Supplemental Table 2). For the 1-year lagged RCL exposure model there was a small decrease in the point estimate, but the confidence intervals remained wide and included the null (PR 1-year lag: 0.85; CI: 0.52, 1.18, Supplemental Table 2). Fourth, when we excluded data from 2020 from the deportation analyses, results were highly consistent (Supplemental Table 3). Lastly when we used a quasi-Poisson specification, the point estimates for the deportation outcomes were slightly attenuated (PR: 0.72; CI: 0.59, 0.84; PR 1-year lag: 0.68; CI: 0.54, 0.82, Supplemental Table 4), but did not change meaningfully.

## Discussion

To the best of our knowledge, this is the first study to empirically examine the relationship between state cannabis law reforms and cannabis-related immigration enforcement. Using repeated cross-sectional data, we tested whether RCL adoption was associated with changes in cannabis-related immigration arrest (between Oct-2014 and May-2018) and deportation (between Jan-2009 and Jun-2020) levels, controlling for secular trends and pre-existing differences between states. Our results suggest that the RCLs were associated with a moderate relative decrease in deportation levels, that was observed relatively consistently across multiple model specifications. Findings also suggested potential relative decreases in immigration arrest levels; however for almost all specifications, associated confidence intervals were wide and included the null. Together these finding support the overall possibility that RCLs may help to mitigate some of the unintended immigration-related consequences of cannabis prohibition. Additional research is needed to replicate and elaborate on these initial results to provide states with the evidence needed to appropriately evaluate the potential costs and benefits of RCL adoption across various public health dimensions.

While our results are specific to immigration arrests and deportations, these findings add to a growing body of literature evaluating the social justice and health equity implications of cannabis law reforms including RCLs. Given significant overlap between drug and immigration enforcement, but relatively few studies on this topic, additional research is needed to examine other important dimensions of these intersecting issues. For example, in this study we examined cannabis policies as a determinant of immigration arrest and deportation. At the same time, a significant body of literature indicates that exposure to aggressive immigration-related enforcement increases risk for multiple adverse consequences, including drug and alcohol misuse [22, 23, 28, 43, 44]. Future research might test relationships between cannabis law reforms and perceived immigration stress to comprehensively evaluate the wider public health consequences of these changing laws.

Future research employing exposures more proximate to immigration enforcement, such as examination of cannabis arrest or conviction rates directly—and related mediation analyses—would also strengthen the evidence for a causal relationship between cannabis policies and immigration enforcement activities. Trends in immigration enforcement should also continue to be monitored as more states adopt RCLs and as additional follow-up time post-legalization is accumulated.

### Limitations

Several limitations of these analyses are noted. First, available arrest data were limited to a 4-year period and thus our analyses may have been underpowered to detect significant changes post-law adoption in RCL compared to non-RCL states. In addition, the inclusion of a 1-year lag further reduced follow-up period, likely contributing to the wider confidence intervals observed for these measures. Beyond the specific limitations of the arrest measure, other limitations of available immigration enforcement data restricted our analyses in multiple ways. Outside of arrests and deportations, we were unable to examine other dimensions of immigration enforcement, such as detentions and detainers, that could be influenced by changing cannabis policy, but which are not currently available. For both the arrest and deportation measures, we had limited access to information on cannabis or other drug related offences, leading to potential undercounting, particularly as we excluded convictions ambiguously referencing ‘marijuana’ or ‘drug possession.’

With respect to our exposure—RCL adoption—we did not examine specific policy dimensions that could be relevant. Studies examining other outcomes of RCLs have, for example, identified heterogeneity in associations by whether or not a state law permits operational dispensaries [45]. Moreover, laws in multiple jurisdictions have legalized cannabis for personal use but maintain prohibitions on commercial sales or other supply mechanisms, and multiple municipalities have adopted their own laws or policies distinct from broader state laws [46]. If these legal distinctions differentially impact cannabis enforcement patterns and related immigration outcomes, our findings may be subject to additional biases. Further research is needed to consider specific facets of RCLs at both state and more local levels.

A third issue is that we were unable to examine potential differences in RCL-associated immigration enforcement by key demographic factors due to limited sociodemographic information and limited variation in outcomes by citizenship. In particular, we did not examine heterogeneity by citizenship, race and ethnicity or indicators of social status. Prior research on arrest disparities has shown that while the absolute numbers of arrests appear to decline in response to RCLs, racialized disparities may persist through multiple pathways. For example, some studies have shown that arrests for public consumption (which typically remains a misdemeanor despite overall state legality) may be more subject to racially and ethnically disparate policing, because there is greater discretion to choose to arrest or issue a warning [8, 9]. Understanding the impact of cannabis law reform on immigration enforcement disparities is an important area for future investigation.

Finally, there were several methodologic limitations of our analysis. First, even though our analytic approach controlled for pre-existing differences between states, changing secular trends in immigration enforcement patterns over time, and time-varying state demographic and sociopolitical factors, unmeasured confounding remains a significant concern. In addition, if immigration-related or other policy changes enacted at the federal level affected states in different ways that we did account for, these unaddressed policy changes could substantially bias our results. Recent methodological literature has highlighted several challenges associated with evaluating policy effects in settings where laws are adopted at multiple timepoints, as with RCLs. In this context, bias could have been introduced if the effects of RCLs changes over time, or differ by the timing of RCL adoption, given that estimated coefficients we present reflect a weighted average over the observation period and across groups [42, 47]. 

## Conclusions

In the past two decades, public support for cannabis law reform has been bolstered by the increasing recognition that criminalization causes substantial harms, especially within communities disproportionately targeted by enforcement activities, including the U.S. non-citizen population. This study adds to emerging evidence documenting the equity-related consequences of cannabis legalization, by showing that state adoption of RCLs likely reduces certain types of immigration enforcement activities. At the same time, arrests and deportations based on cannabis possession convictions remain highly prevalent. To minimize the adverse effects of these trends, it is important that states that have adopted or are considering cannabis legalization take action to ensure that their non-citizens residents are aware of the federal immigration-related consequences of cannabis possession. Further state-level efforts might also concentrate on vacating existing pre-legalization convictions so that past low-level offenses do not continue to jeopardize immigration status.

### Electronic supplementary material

Below is the link to the electronic supplementary material.

Supplementary Material 1

## Data Availability

Data are available from the Transactional Records Access Clearinghouse, Syracuse University. https://trac.syr.edu/immigration/tools/.
