# Supplementary material for: Recreational cannabis legalization and immigration enforcement: a state-level analysis of arrests and deportations in the United States, 2009–2020
Source: BMC Public Health. 2024 Apr 1;24:936. doi: 10.1186/s12889-024-18334-y (PMC10986106; doi:10.1186/s12889-024-18334-y)
Supplement: Supplementary file 1 — Supplementary Material 1 [file 12889_2024_18334_MOESM1_ESM.docx]

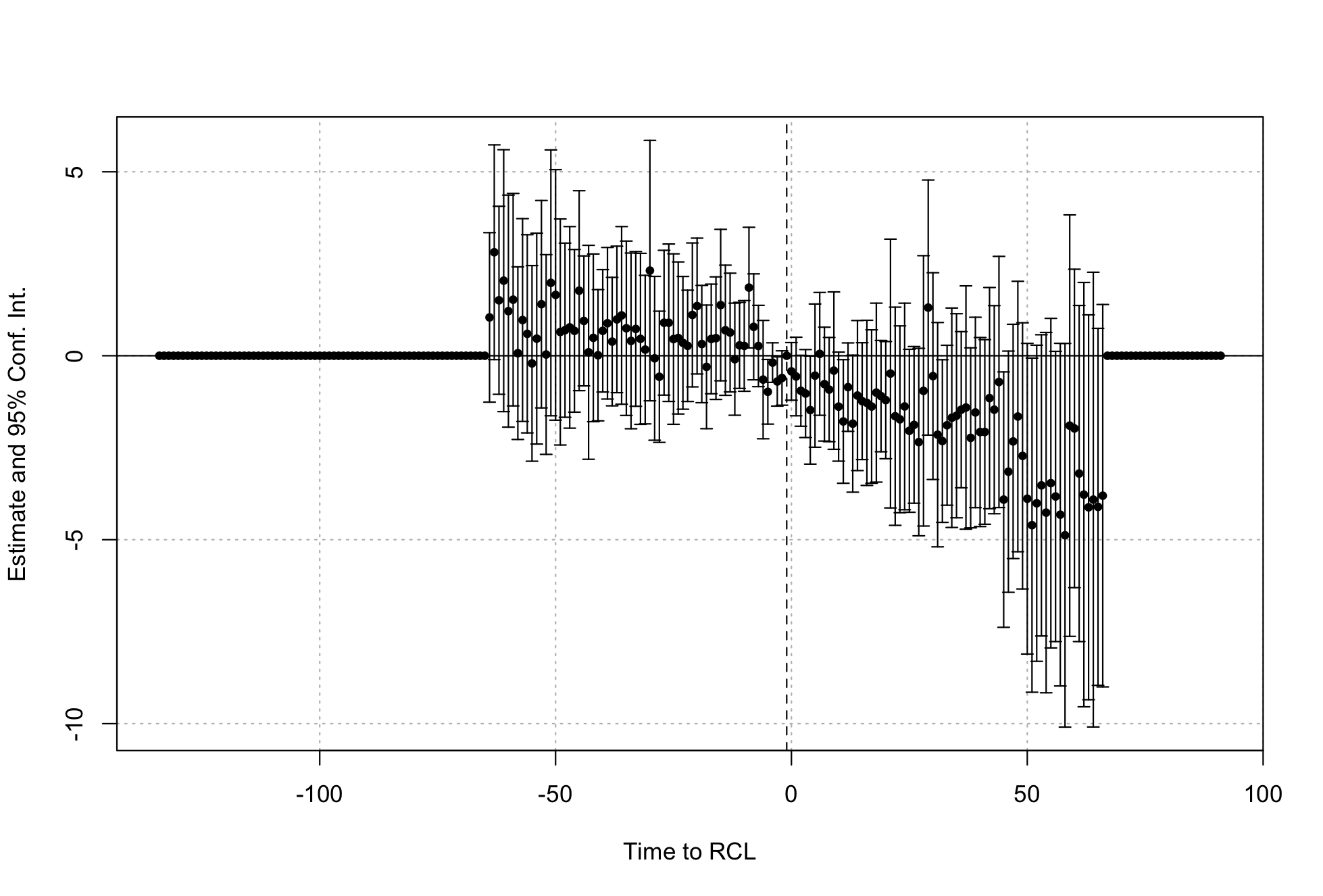


Supplemental Figure 1. Event study of immigration arrests. Recreational cannabis legalization status was operationalized as a binary variable capturing whether a state had a recreational cannabis law in place in the month-year. Models were implemented using a *Poisson* specification and adjusted models included controls for state, month-year, presence of a medical cannabis and decriminalization law, governor’s political party, prevalence of police officers, state median household income, percent of the state population identifying as BIPOC, percentage of the state population not proficient in English and including logged state population as an offset.


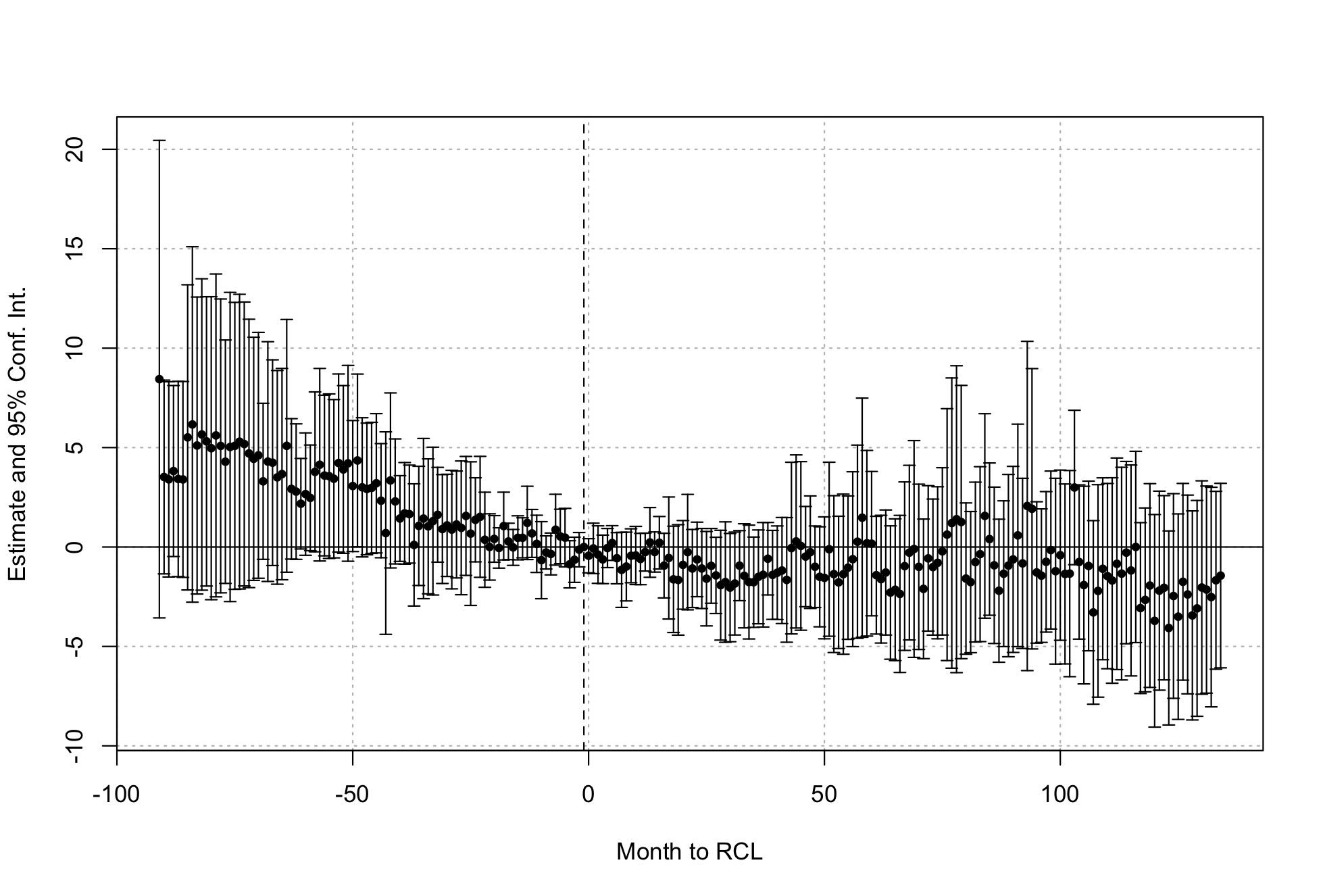


Supplemental Figure 2. Event study of immigration deportations. Recreational cannabis legalization status was operationalized as a binary variable capturing whether a state had a recreational cannabis law in place in the month-year. Models were implemented using a *Poisson* specification and adjusted models included controls for state, month-year, presence of a medical cannabis and decriminalization law, governor’s political party, prevalence of police officers, state median household income, percent of the state population identifying as BIPOC, percentage of the state population not proficient in English and including logged state population as an offset

| Supplemental Table 1. Sensitivity analysis using unauthorized immigrant population estimates, testing associations between recreational cannabis legalization and immigration arrests and deportations related to cannabis possession, 2009-2020 | | | | | | | | | | | | |
| --- | --- | --- | --- | --- | --- | --- | --- | --- | --- | --- | --- | --- |
|  | Cannabis Arrests  (October 2014-May 2018, n=2,244) | | | | | | Cannabis Deportations  (January 2009-June 2020, n=7,004) | | | | | |
|  | Unadjusted | | | Adjusted | | | Unadjusted | | | Adjusted | | |
|  | PR | 95% CI | | PR | 95% CI | | PR | 95% CI | | PR | 95% CI | |
| RCL (immediate) | 0.81 | (0.57, | 1.04) | 0.86 | (0.59, | 1.12) | 0.72 | (0.61, | 0.83) | 0.72 | (0.59, | 0.84) |
| RCL (1-year lag) | 0.86 | (0.56, | 1.17) | 0.91 | (0.58, | 1.23) | 0.78 | (0.65, | 0.90) | 0.71 | (0.57, | 0.85) |
| Recreational cannabis legalization status was operationalized as a binary variable capturing whether a state had a recreational cannabis law in place in the month-year. Models were implemented using a *Poisson* specification and adjusted models included controls for state, month-year, presence of a medical cannabis and decriminalization law, governor’s political party, prevalence of police officers, state median household income, percent of the state population identifying as BIPOC, percentage of the state population not proficient in English and including logged state population as an offset. | | | | | | | | | | | | |

| Supplemental Table 2. Sensitivity analysis excluding always treated states (CO, WA), testing associations between recreational cannabis legalization and immigration arrests and deportations related to cannabis possession, 2009-2020 | | | | | | | |
| --- | --- | --- | --- | --- | --- | --- | --- |
|  | Cannabis Arrests  (October 2014-May 2018, n=2,244) | | | | | |  |
|  | Unadjusted | | | Adjusted | | |  |
|  | PR | 95% CI | | PR | 95% CI | |  |
| RCL (immediate) | 0.75 | (0.52, | 0.98) | 0.84 | (0.56, | 1.11) |  |
| RCL (1-year lag) | 0.83 | (0.56, | 1.13) | 0.85 | (0.52, | 1.18) |  |
| Recreational cannabis legalization status was operationalized as a binary variable capturing whether a state had a recreational cannabis law in place in the month-year. Models were implemented using a *Poisson* specification and adjusted models included controls for state, month-year, presence of a medical cannabis and decriminalization law, governor’s political party, prevalence of police officers, state median household income, percent of the state population identifying as BIPOC, percentage of the state population not proficient in English and including logged state population as an offset. | | | | | | | |

| Supplemental Table 3. Sensitivity analysis excluding 2020, testing associations between recreational cannabis legalization and immigration arrests and deportations related to cannabis possession, 2009-2019 | | | | | | | |
| --- | --- | --- | --- | --- | --- | --- | --- |
|  | Cannabis Deportations  (January 2009-June 2020, n=7,004) | | | | | |  |
|  | Unadjusted | | | Adjusted | | |  |
|  | PR | 95% CI | | PR | 95% CI | |  |
| RCL (immediate) | 0.66 | (0.55, | 0.76) | 0.72 | (0.59, | 0.84) |  |
| RCL (1-year lag) | 0.71 | (0.569 | 0.84) | 0.68 | (0.54, | 0.82) |  |
| Recreational cannabis legalization status was operationalized as a binary variable capturing whether a state had a recreational cannabis law in place in the month-year. Models were implemented using a *Poisson* specification and adjusted models included controls for state, month-year, presence of a medical cannabis and decriminalization law, governor’s political party, prevalence of police officers, state median household income, percent of the state population identifying as BIPOC, percentage of the state population not proficient in English and including logged state population as an offset. | | | | | | | |

| Supplemental Table 4. Sensitivity analysis using quasi-Poisson specifications, testing associations between recreational cannabis legalization and immigration arrests and deportations related to cannabis possession, 2009-2020 | | | | | | | | | | | | |
| --- | --- | --- | --- | --- | --- | --- | --- | --- | --- | --- | --- | --- |
|  | Cannabis Arrests  (October 2014-May 2018, n=2,244) | | | | | | Cannabis Deportations  (January 2009-June 2020, n=7,004) | | | | | |
|  | Unadjusted | | | Adjusted | | | Unadjusted | | | Adjusted | | |
|  | PR | 95% CI | | PR | 95% CI | | PR | 95% CI | | PR | 95% CI | |
| RCL (immediate) | 0.76 | (0.50, | 1.02) | 0.84 | (0.54, | 1.13) | 0.66 | (0.55, | 0.76) | 0.72 | (0.59, | 0.84) |
| RCL (1-year lag) | 0.83 | (0.50, | 1.17) | 0.88 | (0.52, | 1.24) | 0.71 | (0.59, | 0.84) | 0.68 | (0.54, | 0.82) |
| Recreational cannabis legalization status was operationalized as a binary variable capturing whether a state had a recreational cannabis law in place in the month-year. Models were implemented using a quasi-*Poisson* specification and adjusted models included controls for state, month-year, presence of a medical cannabis and decriminalization law, governor’s political party, prevalence of police officers, state median household income, percent of the state population identifying as BIPOC, percentage of the state population not proficient in English and including logged state population as an offset. | | | | | | | | | | | | |
